# Anticancer Potential of Farnesol Against Human Osteosarcoma Saos-2 Cells and Human Colorectal Carcinoma HCT-116 Cells

**DOI:** 10.7759/cureus.49372

**Published:** 2023-11-24

**Authors:** Zakir Hussain Fathima Hinaz, Santhosh Pragya, Devaraj Ezhilarasan, Karthik Shree Harini

**Affiliations:** 1 Dentistry, Saveetha Dental College and Hospitals, Saveetha Institute of Medical and Technical Sciences, Saveetha University, Chennai, IND; 2 Pharmacology, Saveetha Dental College and Hospitals, Saveetha Institute of Medical and Technical Sciences, Saveetha University, Chennai, IND

**Keywords:** colorectal carcinoma, osteosarcoma, cytotoxicity, farnesol, apoptosis

## Abstract

Introduction: Increased colorectal carcinoma (CRC) and osteosarcoma prevalence, low survival rate, poor prognosis, and the limitations of existing anticancer therapies like side effects of drugs, non-specificity, short half-life, etc., pose a need for novel anticancer drugs. Farnesol, an organic sesquiterpene compound, found in the essential oils of various plants has been shown to possess antioxidant, anti-inflammatory, and anticancer properties. However, the anticancer effect of farnesol against CRC and osteosarcoma has not yet been adequately elucidated.

Aim: The aim of the study was to analyze the anticancer effects of farnesol against human osteosarcoma and CRC cell lines.

Materials and methods: Human osteosarcoma (Saos-2) and colorectal carcinoma (HCT-116) cell lines were procured and cultured at 37^o^C and 5% CO_2_. The cells were treated with 10, 20, 40, 60, 80, and 100 µM/ml and 20, 40, 60, 80, 100, and 120 µM/ml of farnesol for 24 hours, respectively. 3-(4,5-dimethylthiazol-2-yl)-2,5 diphenyl tetrazolium bromide assay was performed to assess the cytotoxicity of farnesol on Saos-2 and HCT-116 cells. Acridine orange/ethidium bromide staining was carried out to analyze apoptosis. 4',6-diamidino-2-phenylindole staining was done to observe the nuclear changes. Dichloro-dihydro-fluorescein diacetate staining was performed to assess the farnesol-induced reactive oxygen species (ROS)-mediated cell death.

Results: Farnesol reduced the viability and proliferation of Saos-2 and HCT-116 cells in a dose-dependent manner. Farnesol was able to alter the cellular and nuclear morphology of Saos-2 and HCT-116 cells, promoting cell death. Farnesol-induced apoptosis in human osteosarcoma and colorectal carcinoma cell lines. Early apoptosis was observed in farnesol-treated HCT-116 cells. Additionally, ROS-mediated apoptotic cell death was reported in Saos-2 cells.

Conclusion: Farnesol has the potential to induce cytotoxicity against human osteosarcoma and CRC cell lines.

## Introduction

Osteosarcoma is the most common primary solid cancer of bone and is identified by the development of osteoid and/or immature bone by malignant mesenchymal cells. The incidence of osteosarcoma is 2-3 cases per million across the globe, annually. However, the incidence is higher in adolescents (around 15-19 years of age), attributed to 8-11 cases per million per year [1]. Men are 1.4 times more likely to develop osteosarcoma than females. Different treatment strategies have been suggested and tested against osteosarcoma [1, 2]. Preoperative chemotherapy, surgical excision, pathologic mapping of the resected tumor, and postoperative chemotherapy based on the degree of necrosis of the tumor constitute the standard treatment provided for osteosarcoma patients [1,3]. The main chemotherapeutic agents for osteosarcoma include doxorubicin, adriamycin, cisplatin, methotrexate, cyclophosphamide, and epirubicin. Myelosuppression and gastrointestinal responses, such as anemia, nausea, and vomiting are the most frequent adverse effects of chemotherapy [4]. Cardiotoxicity is the primary adverse effect of anthracycline antibiotics (doxorubicin and epirubicin), and the risk of heart failure rises to 25-30% when adriamycin doses are higher than 550 mg/m^2^ [5]. Cisplatin can also cause kidney damage, hearing loss, hypomagnesemia, and peripheral neuropathy as side effects [6].

Colorectal cancer (CRC) is the third most frequent cancer worldwide. More than 1·2 million patients are diagnosed with CRC every year, and more than 600,000 die from the disease [7]. Risk factors of CRC include improper diet, tobacco smoking, alcohol abuse, and hereditary cancer syndromes. Anticancer drugs like Cetuximab and Bevacizumab have been predominantly used for the treatment of CRC; however, they were reported to cause off-target toxicity [8]. Chemotherapy targets the actively proliferating cancer cells with a potential risk of harming the normal cells [9,10]. They include blood cells, mouth cells, digestive system cells, and hair follicle cells. Therefore, cancer patients are generally advised to be monitored for the rest of their lives for any potential long-term repercussions of their therapies [11].

Collectively, in spite of the progressive cancer treatments like chemotherapy, surgery, radiation therapy, etc., a rise in cancer cases every year worldwide poses a global burden. Therefore, novel anticancer medicines with high efficiency, increased specificity, lower toxicity, and easy accessibility are the current requirements.

Herbal medicines were considered to be an important part of disease treatment from ancient times. It has been reported that about 80% of the world population relies on herbal-based medicines, according to the World Health Organization [12]. Traditional medical systems such as Siddha, Ayurveda, etc. employ varied plants and plant-derived compounds to treat diseases including cancer for their biological properties [13]. Herbal medicines are generally believed to be safe without side effects, undemandingly available, affordable to rural people, and improve the quality of life [14].

One among the plant derivatives is farnesol, an acyclic sesquiterpene alcohol, predominantly found in essential oils of various plants such as citronella, lemon grass, tuberose, etc. [15]. It has been shown to have anti-inflammatory and anticancer properties. It was previously used to suppress allergic asthma, gliosis, and edema. Farnesol was found to alter the expression of several inflammatory mediators such cyclooxygenase-2, inducible nitric oxide synthase, tumor necrosis factor alpha, and interleukin-6 through modulating Ras protein and nuclear factor kappa-light-chain-enhancer of activated B cells activation. Farnesol was demonstrated to have anticancer properties by regulating the tumorigenic proteins and signal transduction cascades in different tumor cell lines in previous studies [15]. Moreover, it has the ability to suppress angiogenesis, cell growth, and survival of cancer cells while inducing apoptosis [16].

Hence, in this study, we have analyzed the cytotoxic effects of farnesol against human osteosarcoma and colorectal carcinoma cell lines. Saos-2 cell line is the commonly used cell line for osteosarcoma studies, which exhibits numerous osteoblastic features [17]. Similarly, the HCT-116 cell line is one of the common cell lines employed in studying the development and progression of colon cancer with respect to proliferation, migration, and invasion [18]. Therefore, the overall objectives of the study were to evaluate the cytotoxic potential of farnesol, to analyze the morphological changes induced by farnesol, and to elucidate the mechanism of farnesol-induced cell death in Saos-2 and HCT-116 cells.

## Materials and methods

Chemicals

Farnesol was procured from Sigma-Aldrich (Burlington, United States). Cell culture essentials like Dulbecco’s modified eagle medium (DMEM) (minimum-low glucose), penicillin, streptomycin, trypsin-EDTA, fetal bovine serum (FBS) and 3-(4,5-dimethylthiazol-2-yl)-2,5-diphenyltetrazolium bromide (MTT) were purchased from GIBCO BRL (Gaithersburg, United States). Other chemicals used in various assays were of analytical grade and purchased locally.

Cell culture

The Saos-2 osteosarcoma cell lines and HCT-116 colorectal carcinoma cell lines were procured from the National Centre for Cell Science (Pune, India) and cultured under 5% CO_2_ and 37^o^C in DMEM with 10% FBS, penicillin (100 units/mL), and streptomycin (100 μg/mL). Saos-2 and HCT-116 cells were treated with different concentrations of farnesol (10, 20, 40, 60, 80, and 100 µM/mL) and (20, 40, 60, 80, 100, and 120 µM/mL), respectively, and incubated for 24 hours.

MTT Assay

Saos-2 cells and HCT-116 cells were seeded in 96-well plates and treated with different concentrations of farnesol for 24 hours. After the treatment, MTT reagent was added and incubated for 4 hours at 37^o^C. The purple formazan crystals were dissolved in dimethyl sulfoxide, and the optical density was measured at 570 nm. Based on the MTT assay, the concentration of farnesol for the subsequent experiments was fixed to be 60 µM/mL [19].

4,6-Diamidino-2-Phenylindole Staining

After treating the Saos-2 and HCT-116 cells with 60 µM/mL of farnesol, nuclear morphology was observed by 4,6-diamidino-2-phenylindole (DAPI) staining, according to Dmitrieva and Burg, 2008. Both control and farnesol-treated cells were fixed in 4% formaldehyde and stained with DAPI, followed by microscopic observation. The staining was performed in triplicates [20].

Acridine Orange/Ethidium Bromide Staining

The farnesol (60 µM/mL) treated HCT-116 cells and control cells were harvested, washed with PBS, and fixed in ethanol. Acridine orange/ethidium bromide (AO/EB) dye mixture was added to the cells. The cells after staining were viewed under a fluorescent microscope and images were captured. The staining was performed in triplicates [21].

Dichlorodihydrofluorescein Diacetate Staining

Farnesol-treated (60 µM/mL) Saos-2 cells and the untreated-control cells were stained with dichlorodihydrofluorescein diacetate (DCFH-DA) which was followed by an incubation at 37^o^C for 30 minutes. The green fluorescence was measured at 485 ± 10 nm and 530 ± 10 nm [22]. The staining was performed in triplicates.

Statistical analysis

Data were reported as mean ± SD. Statistical analysis was performed using IBM SPSS Statistics for Windows, Version 16 (Released 2007; IBM Corp., Armonk, New York, United States). To determine the significance of the difference between the control and treatment groups, the data were subjected to Dunnett’s test and one-way ANOVA. A *p-value*<0.05 was considered significant.

## Results

**Figure 1:**
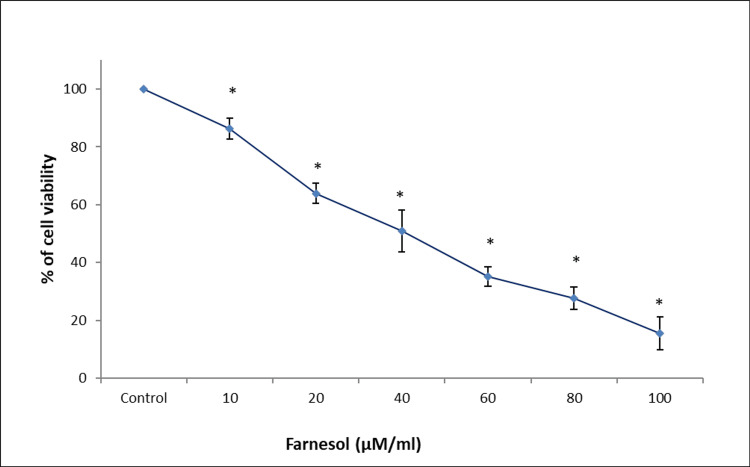
Farnesol treatment suppresses the proliferation of Saos-2 cells A dose-dependent decrease in the proliferation and viability of osteosarcoma cells upon farnesol treatment in 3-(4,5-dimethylthiazol-2-yl)-2,5 diphenyl tetrazolium bromide (MTT) assay. Data are shown as means ± SD (n = 3) compared with the control group, *p < 0.001. Saos-2: human osteosarcoma cell line

**Figure 2:**
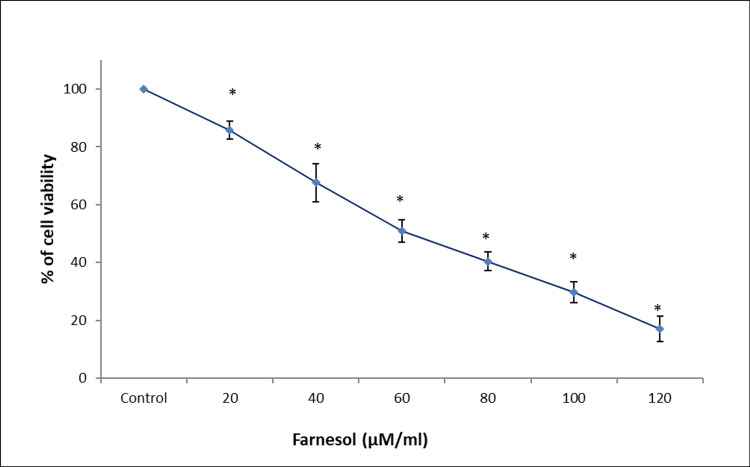
Farnesol treatment suppresses the proliferation of HCT-116 cells A dose-dependent decrease in the proliferation and viability of colorectal carcinoma cells upon farnesol treatment in 3-(4,5-dimethylthiazol-2-yl)-2,5-diphenyltetrazolium bromide (MTT) assay. Data are shown as means ± SD (n = 3) compared with the control group, *p < 0.001. HCT-116: Human colorectal carcinoma cell line

Following a 24-hour treatment with farnesol, the antiproliferative effect of farnesol on osteosarcoma cancer cells and CRC cells was assessed by MTT assay. It was observed that increasing concentrations of farnesol treatment resulted in decreased viability and proliferation of Saos-2 and HCT-116 cells. Maximum cytotoxicity was observed at 100 µM/mL of farnesol in Saos-2 cells and 120 µM/mL of farnesol in HCT-116 cells (Figures 1, 2).

**Figure 3:**
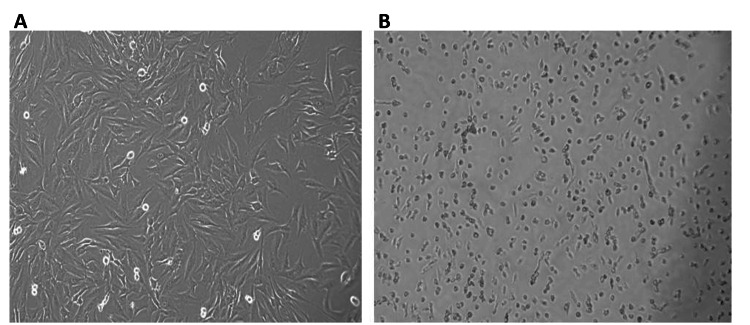
Farnesol alters the morphology of Saos-2 cells (A) The number and morphology of the control Saos-2 cells remained unaltered. (B) Farnesol (60 μM/ml) treatment reduced the number of Saos-2 cells and altered the morphology. Cells were observed under a phase contrast microscope (20x magnification). Saos-2: Human osteosarcoma cell line

**Figure 4:**
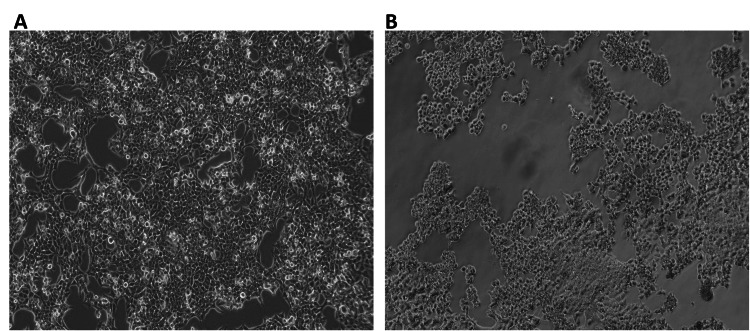
Farnesol alters the morphology of HCT-116 cells (A) The number and morphology of the control HCT-116 cells remained unaltered. (B) Farnesol (60 μM/ml) treatment reduced the number of HCT-116 cells and altered the morphology. Cells were observed under a phase contrast microscope (20x magnification). HCT-116: human colorectal carcinoma cell line

Observation of farnesol-treated Saos-2 and HCT-116 cells under a phase contrast microscope revealed a decrease in the number of cells and altered morphology when compared to the control untreated cells (Figures 3, 4).

**Figure 5:**
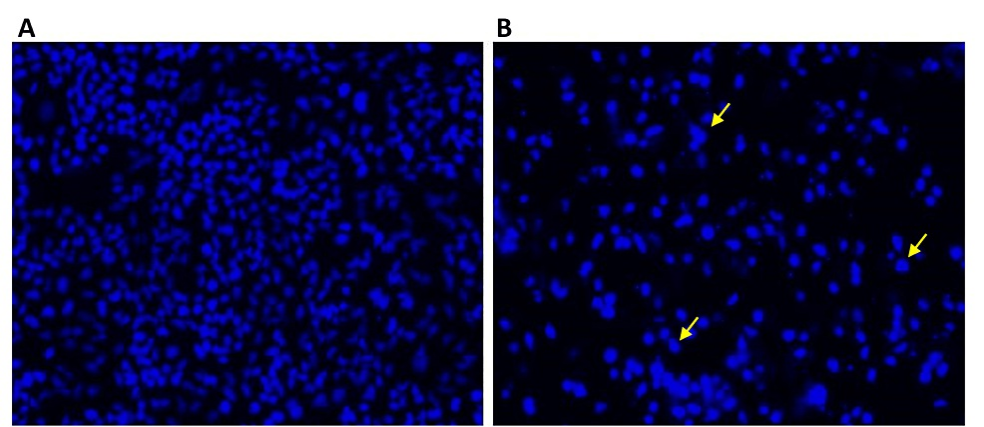
Effect of farnesol on the apoptotic changes of Saos-2 cells (A) Control Saos-2 cells had an intact nucleus. (B) Farnesol (60 μM/ml) treated Saos-2 cells showed condensed and fragmented nuclei. The yellow arrows indicate the nuclear changes in Saos-2 cells. Saos-2: Human osteosarcoma cell line

**Figure 6:**
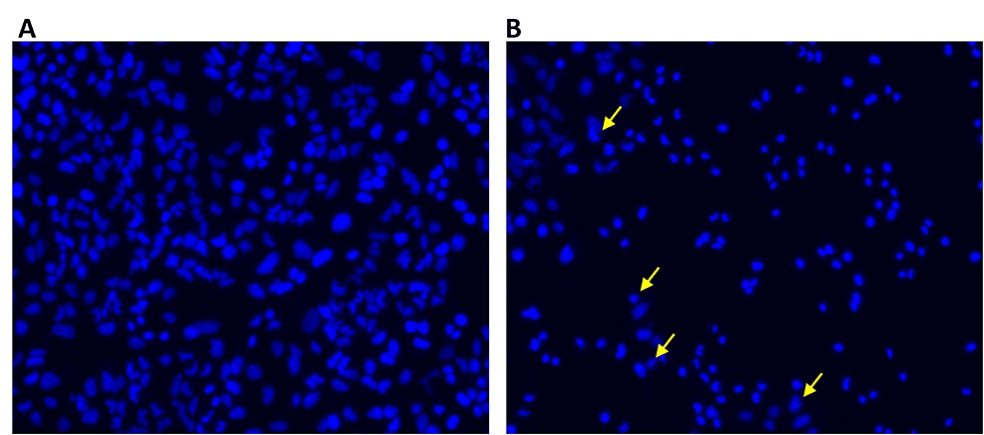
Effect of farnesol on the apoptotic changes of HCT-116 cells (A) Control HCT-116 cells had an intact nucleus. (B) Farnesol (60 μM/ml) treated HCT-116 cells showed condensed and fragmented nuclei. The yellow arrows indicate the nuclear changes in HCT-116 cells. HCT-116: Human colorectal carcinoma cell line

Using DAPI labeling, the apoptotic alterations caused by farnesol were examined. The Saos-2 and HCT-116 cells treated with farnesol had contracted and fragmented nuclei in contrast to the intact nuclear morphology of the control cells. An increase in the number of DAPI-positive cells after farnesol treatment compared to control demonstrated the cytotoxicity of farnesol (Figures 5, 6).

**Figure 7:**
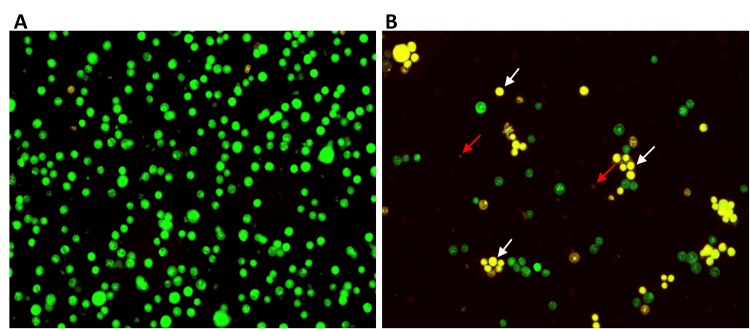
Effect of farnesol in apoptosis of HCT-116 cells Fluorescence imaging with the help of acridine orange/ethidium bromide (AO/EB) staining shows early and late apoptotic cells after 24 hours of farnesol treatment against HCT-116 cells. (A) Control HCT-116 cells were viable. (B) HCT-116 cells treated with 60 μM/ml farnesol showed apoptosis. The white and red arrows indicate early and late apoptotic HCT-116 cells, respectively. HCT-116: Human colorectal carcinoma cell line

To further detect apoptosis in tumor cells, fluorescent imaging using AO/EB was carried out. The green cells indicate normal and viable cells, whereas the yellow and orange cells indicate early and late apoptosis, respectively, and red-colored cells indicate necrotic cells. The findings demonstrate that farnesol is cytotoxic to HCT-116 cells, as evidenced by an increase in the number of early and late apoptotic cells in the treatment group compared to the control group (Figure 7).

**Figure 8:**
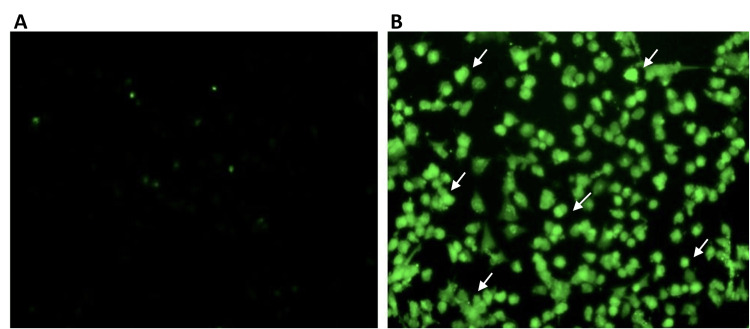
Effect of farnesol in ROS-induced apoptosis of Saos-2 cells Fluorescence imaging with the 2, 7-dichlorodihydrofluorescein diacetate (DCFH-DA) staining increased the accumulation of ROS in Saos-2 cells after 24 hours of farnesol treatment. (A) Control Saos-2 cells. (B) Saos-2 cells treated with 60 μM/ml farnesol showed ROS accumulation. The white arrows indicate ROS accumulated Saos-2 cells. Saos-2: Human osteosarcoma cell line; ROS: Reactive oxygen species

To demonstrate the mechanism of farnesol-induced apoptosis, a DCFH-DA assay was performed. The results indicated that increased green fluorescence upon farnesol treatment represents elevated intracellular oxidative stress in Saos-2 cells due to the accumulation of reactive oxygen species (Figure 8).

## Discussion

To address the limitations of conventional anticancer medications and therapies, several novel anticancer drugs, plants, and plant compounds have been studied in recent decades. Among these, natural plant-based products have gained a great interest in developing anticancer drugs. The presence of components such as flavonoids, vinca alkaloids, diterpenes, sesquiterpenes, tannins, etc. in the plants is responsible for their anticancer activities [23]. Plant derivatives like curcumin (polyphenol), silibinin (flavonolignan), eugenol (alkaloid), Hederoside C (triterpene), and coleusin factor (diterpenoid) were reported to suppress osteosarcoma [24]. Similarly, halocynthiaxanthin (carotenoid), luteolin (flavonoid), curcumin (polyphenol), sesamol (phenolic compound), resveratrol (polyphenol), ginsenosides (triterpenoid saponin), etc. were effective in preventing the development and progression of colorectal cancer, according to earlier studies [25].

Likewise, farnesol (trans, trans-3,7,11-trimethyl-2,6,10-dodecatriene-1-ol) is one of the plant compounds, recognized for its medicinal properties. The antimicrobial, antifungal, antibiofilm, antitumor, and anticancer properties of farnesol have been investigated in several in vitro and in vivo studies previously [26]. Farnesol has been studied in several tumor cell lines, including the prostate, breast, lung, pancreas, cervical cancers, oral squamous cell carcinoma, meningioma, multiple myeloma, and T lymphoblastic leukemia cells [27]. Farnesol has been shown to suppress cancer cell proliferation, induce apoptosis, and prevent angiogenesis. In an in vitro study, farnesol was observed to suppress the proliferation and growth of Caco-2 (adenocarcinoma) and A549 (lung cancer) cell lines. Moreover, farnesol promoted the growth of normal human lung epithelial BEAS-2B cell line. This denotes that farnesol possesses cancer cell-specific targeting ability [27]. Similarly, our study also showed that farnesol was able to significantly reduce the viability of Saos-2 cells and HCT-116 cells in a dose-dependent manner. The reduction in the proliferation and survival of cancer cells upon farnesol treatment could be attributed to the cytotoxic property of farnesol against Saos-2 and HCT-116 cells.

The results of the MTT assay and microscopic analysis revealed that farnesol has a potent antiproliferative property. Further, to elucidate the mechanism of farnesol-induced cell death, DAPI, AO/EB, and DCFH-DA staining were performed. Apoptosis is one of the important mechanisms of cell death caused by anticancer agents [28]. It is a regulated cascade that inhibits the survival and growth of abnormally proliferating cancer cells. Apoptosis is generally classified into two types intrinsic and extrinsic apoptosis. The hallmarks of apoptosis are reported to be altered morphological structure, shrinkage of cells, disrupted cell membrane, condensed and fragmented nuclei, change in the oxidative milieu, and aberrated cellular signaling pathways. In a previous study, chitosan-encapsulated nickel oxide, tin dioxide, and farnesol nanoparticles induced nuclear changes in the breast cancer MDA-MB-231 line [29]. Consistently, DAPI staining in our study also showed fragmented and condensed nuclei in Saos-2 and HCT-116 cells, leading to apoptosis. To confirm farnesol-induced apoptosis, AO/EB staining was also performed. The principle behind AO/EB staining is that acridine orange enters both the live and dead cells, producing green fluorescence in the living cells. Whereas only dead cells with compromised cell membrane allows ethidium bromide to enter the cells producing a yellow to red fluorescence. Farnesol was demonstrated to induce early and late apoptosis in leukemia MOLT-4 cells and in HeLa cervical cancer cells indicated by increased yellow and orange fluorescence [9,30]. In accordance, the present study also exhibited the apoptotic property of farnesol against HCT-116 cells by promoting early and late apoptosis. However, in our study farnesol was observed to significantly promote early apoptosis than late apoptosis. Increased production of ROS creates oxidative stress in the cancer cells which ultimately results in apoptosis. Elevated levels of ROS induce the activation of the mitochondria-dependent pathway of apoptosis. Farnesol was previously reported to induce ROS production in leukemia MOLT-4 cells, which led to a decrease in cell viability [9]. Correspondingly, increased production of ROS in Saos-2 cells was evidenced by the DCFH-DA assay in the current study.

To summarize, farnesol exhibited cytotoxicity against Saos-2 and HCT-116 cells by inducing ROS-mediated apoptosis and it could be a potential anticancer drug in the near future with additional extensive studies.

Limitations

This research work elucidates the cytotoxic potential of farnesol against Saos-2 and HCT-116 cells. However, the specificity of farnesol on cancer cells without affecting the normal cells has not been studied currently. Analyzing apoptosis and cell cycle would provide additional insight. Also, the pharmacokinetics and pharmacodynamics aspects must be evaluated. Provided, the safety of this novel compound must be tested for an efficient anticancer treatment against osteosarcoma and CRC.

## Conclusions

The findings indicate that farnesol possesses significant antiproliferative and apoptotic effects against Saos-2 cells and HCT-116 cells in a concentration-dependent manner. This study imparts a base for detailed in vivo studies of farnesol as a potent anticancer drug for osteosarcoma and CRC.
